# Effect of Silica- and Cellulose-Based Nanofillers in Poly(butylene succinate-co-butylene adipate)-Based Composites

**DOI:** 10.3390/polym18020189

**Published:** 2026-01-09

**Authors:** Camilla Ferretti, Miriam Cappello, Patrizia Cinelli, Damiano Rossi, Nicolas Sbirrazzuoli, Giovanna Molinari, Maria Cristina Righetti, Maurizia Seggiani

**Affiliations:** 1 Department of Civil and Industrial Engineering, University of Pisa, Largo Lazzarino 1, 56122 Pisa, Italy; c.ferretti17@studenti.unipi.it (C.F.); miriam.cappello@unipi.it (M.C.); damiano.rossi@unipi.it (D.R.); maurizia.seggiani@unipi.it (M.S.); 2 Institute of Chemistry of Nice, UMR CNRS 7272, University Côte d’Azur, 06100 Nice, France; nicolas.sbirrazzuoli@univ-cotedazur.fr; 3 Institute for Chemical and Physical Processes (IPCF), National Research Council (CNR), Via Moruzzi 1, 56124 Pisa, Italy; giovanna.molinari@pi.ipcf.cnr.it (G.M.); mariacristina.righetti@cnr.it (M.C.R.)

**Keywords:** poly(butylene succinate-co-butylene adipate), cellulose, silica, nanocomposites, thermal, mechanical, barrier properties

## Abstract

In an effort to reduce global dependence on fossil-based polymers and advance toward a more sustainable materials industry, research over recent decades has increasingly focused on the development of bio-based polymers and broadening their potential applications. Within this context, the present study investigates nanocomposites based on poly(butylene succinate-co-butylene adipate) (PBSA), reinforced with two types of nanofillers: silicon dioxide nanoparticles (SiO_2_ NPs) and cellulose nanofibrils (CNFs). The main objective of this work is to examine how the morphology, geometry, and chemical nature of the nanofillers influence the thermal, mechanical, and barrier properties of PBSA, as well as its biodegradability. For each nanofiller, three formulations were prepared, containing 1, 2, and 5 wt% of filler, respectively. Scanning electron microscopy (SEM) analysis confirmed good dispersion and minimal aggregation in the SiO_2_-based systems, whereas marked aggregation was observed in the CNF-based samples. Thermal analysis indicated that the intrinsic thermal properties of neat PBSA were largely preserved. Mechanical testing revealed improvements in both the elastic modulus and elongation at break for most nanocomposite samples. In particular, CNFs provided the most consistent reinforcing effect, with enhancements of approximately 40% in the elastic modulus (495.4 vs. 356.4 GPa in neat PBSA) and 52% in elongation at the break (185.1 vs. 122.0% in neat PBSA) with 5 wt% loading. Additionally, the incorporation of nanofillers did not alter the surface hydrophilicity, but it did improve the oxygen barrier performance and enhanced disintegration under composting conditions. Overall, these findings demonstrate the promising potential of PBSA-based nanocomposites for sustainable rigid packaging applications.

## 1. Introduction

Conventional plastics are major contributors to greenhouse gas emissions and persistent environmental pollution, representing a growing risk due to microplastic formation [1]. If current trends persist, plastic-related emissions are projected to reach 1.3 Gt annually by 2030, accounting for a substantial portion of the remaining global carbon budget [2]. Biodegradable and bio-based polymers offer a promising route to reduce environmental impacts. Among these polymers aliphatic bio-polyesters such as poly(butylene succinate-co-butylene adipate) (PBSA) have gained attention for their biodegradability in diverse environments and their potential to replace fossil-based plastics in single-use applications. Thus PBSA, a partially bio-based co-polyester, combines favourable mechanical properties with the ability to degrade in soil, marine water, and both home and industrial composting conditions [3,4,5,6,7]. However, as for many bioplastics, the practical applications of PBSA are limited by certain drawbacks, such as poor mechanical properties in terms of strength and modulus, poor thermal stability, and poor barrier properties compared to conventional plastics. To overcome these limitations, the incorporation of nanofillers has emerged as an effective strategy to enhance the performance of biodegradable polymers [8].

Nanofillers are commonly classified according to the number of dimensions in the nanometric range. Isodimensional nanoparticles (NPs), with dimensions below 100 nm, are defined as zero-dimensional (0D) materials, whereas nanofillers with one or two dimensions exceeding the nanoscale are classified as one-dimensional (1D), such as nanofibers, nanotubes, nanorods, and whiskers, or two-dimensional (2D), including nanoplatelets and nanoflakes. Nanofillers’ morphologies strongly affect the composite properties of materials made with them [9,10,11,12]. Although their high surface area promotes extensive interfacial interactions and efficient load transfer at low filler contents, performance depends not only on dimensionality but also on filler size, morphology, and chemical nature. Composites combining a bio-based and/or biodegradable polymer matrix with fillers having at least one nanoscale dimension are generally referred to as bionanocomposites [13]. Various nanofillers have been combined with biodegradable polymeric matrices, including graphene [14], organically modified layered silicates [15,16], carbon nanotubes [17], nanosilica [18,19,20,21,22,23,24,25,26], and nanocellulose [27,28,29,30,31,32,33,34].

Over the past decade, SiO_2_ nanoparticles have been widely investigated to reduce oxygen and water vapor permeability in packaging materials suitable also for electronics, and medical devices packaging [35]. Improved barrier properties have been reported for several biodegradable polymers including poly(3-hydroxybutyrate--3-hydroxyvalerate) (PHBV) [19,23], poly(butylene adipate-co-) (PBAT) [21], poly(L-lactic acid) PLLA/PHBV blends poly(lactic acid) (PLA) [24,25], and poly(3-hydroxybutyrate--3-hydroxyhexanoate) (PHBH) [26]. In many cases, enhancements in tensile strength, elongation at break, toughness, and thermal stability were also observed, although these are strongly dependent on nanosilica content and surface functionality [18]. Koreshkov [19] reported increased barrier properties, toughness, and biodegradation rate in PLLA/PHBV blends reinforced with SiO_2_ nanoparticles modified with L-lactic acid oligomers. Similar improvements were observed by Venkatesan and Rajeswari [21] in PBAT/SiO_2_ nanocomposites, which also exhibited antimicrobial activity. Ojha and Das [23] showed that PHBV reinforced with SiO_2_ nanoparticles displayed increased elastic modulus (E), yield stress (σy), elongation at break (εb), and impact strength, with optimal performance at filler contents between 1 and 1.5 wt%. When a strong enhancement of mechanical performance is required, 1D nanofillers are often preferred due to their extended geometry, which promotes effective stress transfer between matrix and filler [36]. Hashim et al. [37] formulated bionanocomposites having PBS as matrix material rein-forced with nanofibrillated bacterial cellulose (NFBC), combined with hydroxypropyl cellulose (HPC) as dispersing agent, and found an increase in flexural elastic modulus in melt-kneaded nanocomposites for all concentrations of the nanofiller (0.25, 1, 2, and 5 wt%) as well as an increase in flexural ultimate strength for all formulations apart for the one containing 5 wt% HP-NFBC, probably due to the agglomeration of the filler. Thus, cellulose nanofibers or nanofibrils (CNFs) have been commonly selected to reinforce biodegradable polymers for sustainable packaging applications. Several authors have observed greater mechanical properties in CNF-reinforced poly(butylene succinate) (PBS) nanocomposites [31,34]. Platnieks et al. [28] registered a storage modulus two times higher than that of pure PBS when 40 wt% of CNFs was incorporated in the matrix. Greater tensile strengths and elastic modulus, at the expense of the elongation at break, have been reported by Cindradewi et al. [34] in bionanocomposites with PBS as matrix material; these were reinforced with nanofibrillated bacterial cellulose (NFBC), combined with hydroxypropyl cellulose (HPC) as dispersing agent. The authors found an increase in flexural elastic modulus in melt-kneaded nanocomposites for all concentrations of the nanofiller (0.25, 1, 2, and 5 wt%) as well as an increase in flexural ultimate strength for all formulations apart for the one containing 5 wt% HP-NFBC. This was probably due to agglomeration of the filler. CNFs were also investigated in combination with PBAT as biodegradable polymeric matrix. Avella et al. [32] exhibited an increase in the Young’s Modulus while maintaining a high deformability. Analogous results have been reported by Zhou et al. [31]. Additionally, CNF inclusion has shown to enhance biodegradation and disintegration rates in PLA-based nanocomposites [29] being natural fillers easily degradable. Authors have also noted enhanced barrier to oxygen properties in CNF-reinforced PBAT [30,33].

In this context, the present study investigates PBSA-based nanocomposites reinforced with spherical silica nanoparticles (SiO_2_ NPs) and cellulose nanofibrils (CNFs), selected to enhance gas barrier and mechanical properties, respectively. The objective is to improve mechanical strength, thermal stability, and barrier performance without compromising PBSA biodegradability and overall material sustainability.

Two series of nanocomposites were prepared, incorporating different amounts of nanofiller (1, 2, and 5 wt%), SiO_2_ NPs, or CNFs in a PBSA matrix by melt-compounding. The raw PBSA analysis was used to study the thermal behaviour of the obtained materials. The thermomechanical properties were evaluated by dynamic mechanical thermal analysis (DMTA). By choosing not to use compatibilizers in the formulation of the nanocomposites, the study aims to understand the intrinsic interfacial interactions between PBSA and the nanofillers. In addition, the oxygen permeability, surface hydrophobicity/hydrophilicity, and disintegration behaviour of the produced PBSA nanocomposites were evaluated under composting conditions to assess the feasibility of these nanosystems as sustainable single-use materials in packaging applications, for example.

## 2. Materials and Methods

### 2.1. Materials

The polymer matrix used in this study was BioPBS™ FD92PM (Mitsubishi Chemical, Tokyo, Japan), a partially bio-based grade of PBSA. This material is certified for biodegradability and compostability under various international standards and exhibits suitable mechanical properties for several practical applications, such as in packaging and agriculture; for these reasons, a better understanding of the effect of the addition of nanofillers, and their shape and amount, in this extremely interesting polymeric matrix is of value. Two types of nanofillers were selected to reinforce the PBSA matrix:

- Silica nanoparticles (SiO_2_ NPs): Evonik AEROSIL^®^ R 972 (Evonik, Essen, Germany), an amorphous fumed silica that has been surface-treated with dimethyldichlorosilane to enhance hydrophobicity and dispersion, with characteristic dimension ranging between 2.5 and 50 nm [38].
- Cellulose nanofibrils (CNFs): supplied by Nanografi (Çankaya/Ankara, Turkey) (SKU: NG01NC0201), derived from cotton, and carboxymethylated to be rendered more hydrophilic, with dimensions of 10–20 nm in width and 2–3 µm in length as per the datasheet in [39].

#### 2.1.1. Masterbatch Preparation and Blending

To ensure accurate dispersion and dosing of nanofillers, two masterbatches were pre-formulated by a third-party supplier (Frilvam S.p.A., Nerviano, Italy) with a double-screw extruder, 20 mm, L/D 40, operating in the 135–145 °C range at 50 rpm, with a rate of 2.0 Kh/h.

- SiO_2_ NPs masterbatch: containing 10 wt% fumed silica nanoparticles in the PBSA.
- CNFs masterbatch: containing 20 wt% carboxymethylated cellulose nanofibrils in the PBSA.

These masterbatches were manually blended with the plain PBSA to achieve final nanofiller concentrations, as discussed below. Prior to extrusion, all materials were dried under vacuum at 50 °C for 24 h to minimize moisture content.

#### 2.1.2. Extrusion Process and Parameters

PBSA-based nanocomposites were prepared using a Brabender KE 19/25 single-screw extruder (Anton Paar, Graz, Austria) with a 19 mm diameter screw.

Two series of formulations were developed:

- PBSA–SiO_2_ nanocomposites: prepared by blending PBSA with a 10 wt% masterbatch of SiO_2_ NPs to achieve final filler contents of 1, 2, and 5 wt%.
- PBSA–CNF nanocomposites: prepared using a 20 wt% masterbatch of CNFs to obtain final filler contents of 1, 2, and 5 wt%.

These filler content values (1, 2, and 5 wt%) were chosen as they fall within the typical range for similar nanocomposite systems reported in the literature on different polymeric matrices [40]. Moreover, at this stage of the study, no compatibilizers were used, supporting the investigation of intrinsic interfacial interactions between the matrix and filler phases without interference from any other additive. The extrusion was carried out at a screw speed of 50–55 RPM, with a temperature profile optimized for PBSA processing:

- SiO_2_-based formulations: the barrel temperatures ranged from 133 °C (feed zone) to 142 °C (die).
- CNF-based formulations: slightly higher barrel temperatures were used (135–145 °C) to accommodate the higher viscosity and moisture sensitivity of cellulose-reinforced blends.

The extrudates were cooled in a water bath and pelletized. Notably, the CNF-based formulations exhibited a distinct water-dragging behaviour during the cooling step: the filaments retained and transported water along the bath, forming a gelatinous layer on the surface. This phenomenon, more pronounced at higher CNF loadings, was attributed to the hydrophilic nature of cellulose and sub-optimal dispersion, leading to partial swelling or gelation of the nanofibrils. To remove the residual moisture, the pellets were post-dried in a ventilated oven with specific, tailored conditions:

- SiO_2_-based nanocomposites: conditioned at 40 °C for approximately 24 h.
- CNF-based nanocomposites: a two-step conditioning protocol was employed, consisting of a first step at 60 °C for 1 h followed by 40 °C for 48 h.

All dried samples were vacuum-sealed to prevent the reabsorption of moisture prior to experimental analysis. The six nanocomposite systems obtained via extrusion compounding were tested alongside the neat PBSA matrix. In order to ensure consistent thermal history across all the tested formulations, pure PBSA was subjected to an extrusion process that was carried out using similar parameters to those employed in the extrusion process of the nanocomposite systems. All formulations evaluated in this study were assigned abbreviated codes for clarity. The neat PBSA sample was labelled as PBSA, while nanocomposites reinforced with SiO_2_ NPs at 1, 2, and 5 wt% were denoted as P1S, P2S, and P5S, respectively. Similarly, formulations containing CNFs at the same loading levels were labelled as P1C, P2C, and P5C.

### 2.2. Film Production

Compression-moulded films were produced using a Noselab ATS 10T press (Noselab ATS, Bovisio Masciago, Italy). Approximately 3 g of granules were pressed between Teflon sheets at 175 °C and 400 bar for 2 min. Film thicknesses ranged from 0.084 mm to 0.102 mm.

### 2.3. Characterization Methods

#### 2.3.1. Morphological Analysis

Scanning electron microscopy (SEM) was performed using a FEI Quanta 450 FEG (FEI, Hillsboro, OR, USA) to examine filler dispersion and interfacial morphology. Samples were cryo-fractured and sputter-coated with platinum prior to imaging.

#### 2.3.2. Thermal Characterization

Thermal properties were assessed using differential scanning calorimetry (DSC) and thermogravimetric analysis (TGA):

- Differential Scanning Calorimetry

Two thermal programmes were employed, one to evaluate the overall thermal profile (hereby referred to as DSC1) and the other to specifically investigate the glass transition temperature (hereby referred to as DSC2). The analyses were conducted using Perkin Elmer DSC 6000 and Perkin Elmer DSC 8500, respectively. As for the DSC1 program (Pyris version 13.4, 2020 Perkin Elmer, Waltham, MA, USA), about 10 mg of samples were equilibrated at 20 °C for 2 min and then cooled from 20 °C to −60 °C. Then, samples were heated from −60 to 120 °C, maintained at 120 °C for 3 min, cooled from 120 to −60 °C, and subjected to a second run heating from −60 to 120 °C. The analysis was carried out using a heating/cooling rate of 10 °C/min, under a nitrogen flow of 20 mL/min. The temperature range used in the DSC1 was not sufficient to evaluate the glass transition temperature of the investigated materials; thus, a second campaign of analysis was carried out. According to the DSC2 program, samples were first equilibrated at −70 °C and kept at this temperature for 2 min to reach thermal stabilization. Then, a first heating step was performed, under nitrogen (20 mL/min), from −70 to 140 °C, increasing with a constant rate of 10 °C/min.

The degree of crystallinity, determined as the weight fraction of the crystalline domains over the whole material, was calculated from the DSC1 data via the following expression (Equation (1)):

(1) $$X_{c}\left(\%\right)=\frac{\Delta H_{m}\left(T_{m}\right)}{\Delta H_{m}^{0}(T_{m}^{0})}\times 100$$

where $\Delta H_{m}(T_{m})$ is the enthalpy of fusion measured at the melting point, $T_{m}$, and $\Delta H_{m}^{0}(T_{m}^{0})$ is the melting enthalpy of the (ideal) purely crystalline polymer measured at the equilibrium melting point, $T_{m}^{0}$. The value for $\Delta H_{m}^{0}(T_{m}^{0})$ was set to 200 J/g [41].

- Thermogravimetric Analysis

Thermal stability was measured using a Netzsch STA 2500 Regulus (Selb, Germany) under nitrogen flow, from 25 °C to 800 °C at 10 °C/min.

#### 2.3.3. Mechanical Testing

- Tensile tests: conducted on injection-moulded dog-bone specimens (5 samples per formulation) using a Galdabini Quasar 10 machine (Galdabini, Cardano al Campo, Italy), following ISO 527 standards [42].
- Dynamic mechanical thermal analysis (DMTA): performed on rectangular specimens using a Netzsch DMA 303 Eplexor to evaluate viscoelastic properties over a temperature range of –70 to 60 °C; tests were performed in tensile loading mode at a frequency of 1 Hz and a fixed deformation amplitude of 70 μm, using samples 20 mm in length with a cross-sectional area of 5 × 2 mm.

#### 2.3.4. Other Characterizations

- Water contact angle (WCA): contact angle measurements were carried out at room temperature by using a DSA25S Drop Shape Analyzer (Krüss Scientific, Hamburg, Germany). Tests were performed by using the sessile drop method: a 5 μL droplet of distilled water was placed on the surface of the samples (5 measurements for sample). Images were acquired with a high-resolution camera, and the data were analysed by Kruss ADVANCE 1.14 Software for drop shape.
- Oxygen permeability (OP): tests were performed using a PermeO_2_ Gas Permeability Tester (ExtraSolution, Pieve Fosciana, Italy) under anhydrous conditions at 23 °C, following ISO 15105-2 [43].
- Disintegration under composting conditions: assessed according to ISO 20200 [44] using synthetic solid waste in aerobic conditions at 58 °C. Only P5S, P5C, and PBSA were tested as representative samples of the series.

## 3. Results

### 3.1. Nanocomposites Morphology

**Figure 1:**
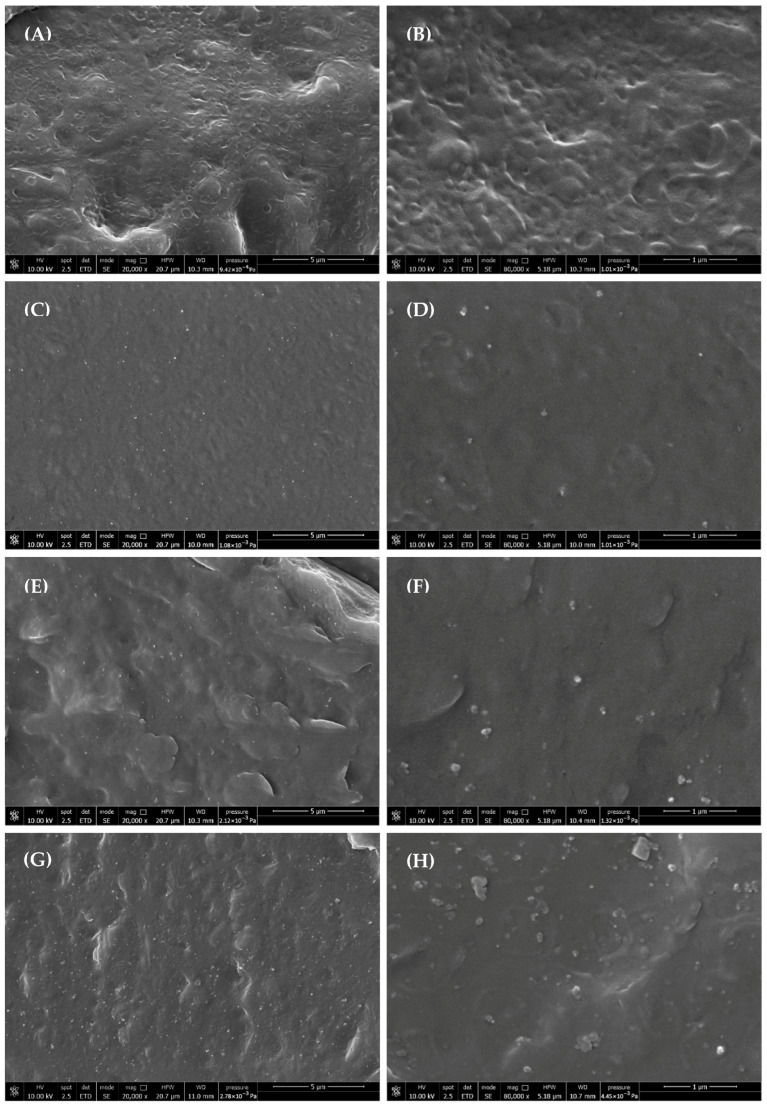
SEM images of (**A**,**B**) PBSA, (**C**,**D**) P1S, (**E**,**F**) P2S, and (**G**,**H**) P5S, surface samples.

SEM imaging confirmed good dispersion of SiO_2_ nanoparticles (Figure 1), with minimal aggregation up to 2 wt%. While at 5 wt%, larger clusters of filler were observed, but the maximum observed size never exceeded 280 nm. Since the size of the NPs is reported by the supplier datasheet to range between 2.5 and 50 nm and considering the absence of a compatibilizer in the nanocomposite formulations, this value is to be considered satisfactory.

**Figure 2:**
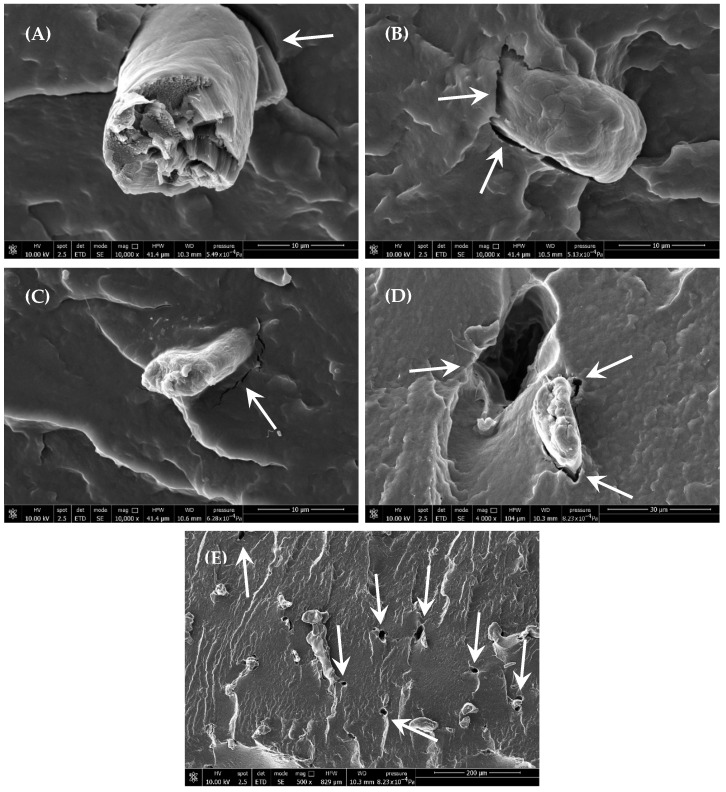
SEM images of (**A**,**B**) P1C, (**C**) P2C, and (**D**,**E**) P5C fractures in liquid nitrogen, arrows evidence the voids of fibres pullout.

On the other hand, as observed in fractured samples, CNFs tended to form microfibrillar aggregates (Figure 2), deviating from their nominal nanoscale dimensions (10–20 nm). These aggregates exhibited poor interfacial adhesion, revealed by both the presence of cracks at the interface between filler and matrix and by pull-out of the fibres (indicated by arrows in the SEM images) on the fracture surfaces, as well as the presence of voids at the interface, suggesting limited compatibility with the PBSA matrix.

### 3.2. Thermal Properties

**Figure 3:**
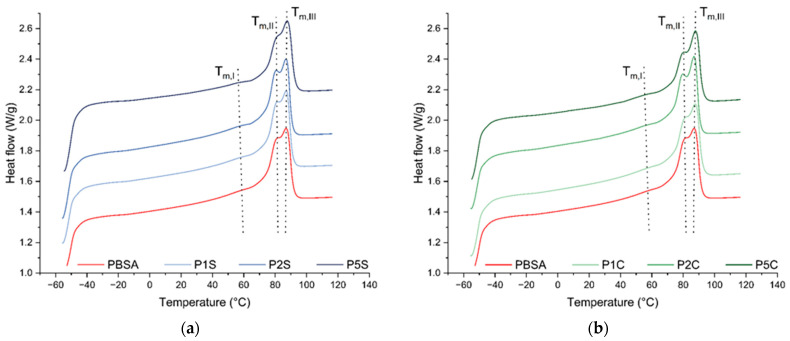
Second heating curves obtained via DSC1 investigation of (**a**) SiO2-reinforced nanocomposites compared to neat PBSA and of (**b**) CNFs-reinforced nanocomposites compared to neat PBSA.

The DSC results revealed that the incorporation of both SiO_2_ and CNFs did not significantly alter the melting temperature ($T_{m}$), crystallization temperature ($T_{c}$), degree of crystallization ($X_{c}$) of PBSA (Figure 3, Table 1), or the glass transition temperature ($T_{g}$) (Table 2). As a result of the DSC1 investigation, it was found that all formulations exhibited a multimodal melting behaviour, with a secondary, broad melting peak at a lower temperature and a double melting peak at a higher temperature (Figure 3). This complex melting behaviour is attributable to the typical melting–recrystallization–remelting process that may occur upon heating for PBSA, with the development of crystalline fractions with different levels of perfection, as already observed and described in detail for the homopolymer PBS.

This thermal behaviour, characterized by isodimorphism, is consistent with a PBSA with a weight ratio of the two monomers (butylene succinate, BS, and butylene adipate, BA) of about 80:20 (BS:BA), as can be concluded by comparing the results of the thermal investigation with studies in the literature that explore the effect of comonomer ratio on the PBSA thermal behaviour [45,46]. According to studies by Pérez-Camargo et al. and Debuissy et al., with PBSA having a compositional ratio of 80:20, during crystallization from melt state, PBS-type crystals are formed with partial inclusion of BA units develop [45,46,47]. In light of this, the bi-modal melting peak located between 80 and 90 °C, indicated as $T_{m,II}$ and $T_{m,III}$ (Table 1), can be attributed to the superposition of two distinct thermal events, which have already been observed in the melting behaviour of pure PBS: the reorganization and thickening of existing lamellae, and a melt–recrystallization–remelt process. This dual melting behaviour at higher temperatures allows us to conclude that two crystalline fractions are present in the material: the initial lamellar structures and a more ordered phase formed via lamellar thickening and structural rearrangement [48,49], whereas the minor endothermic event present around 60 °C, indicated as $T_{m,I}$ (Table 1), is to be ascribed to the melting of weakly ordered PBS crystals that incorporate a high amount of BA units, leading to a depression in the value of the melting temperature. Nonetheless, the calculated $X_{c}$ values (which were obtained by taking into consideration all three endothermic events) are approximately constant and within the bounds of experimental uncertainty. All the results obtained from the DSC1 investigations are summarized in Table 1.

Table 2 reports the glass-transition temperatures of the samples obtained by DSC2 program measurements. As previously mentioned, $T_{g}$ did not show any significant variations in the nanoreinforced formulations if compared to the neat polymer matrix, suggesting that none of the fillers is able to affect the molecular mobility of PBSA. The values obtained via thermal investigation employing the DSC2 program are compared in Table 2, with those acquired via DMTA from the peak of the loss modulus curve. Despite the fact that, the two techniques probe different aspects of the glass transition (DSC measures changes in heat capacity, while DMTA assesses molecular mobility), it can be appreciated that the two sets of data do not substantially differ from one another. Most importantly, in both sets, the variation in $T_{g}$ recorded across all samples does not exceed 1.5 °C; this is a range which can be considered as being within the experimental variability typical of DSC analyses.

**Figure 4:**
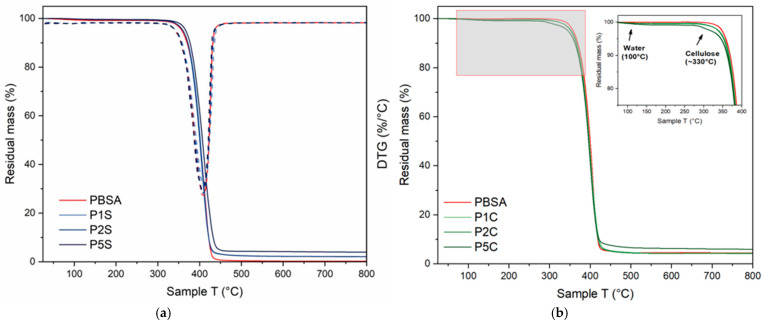
TGA curves of (**a**) SiO_2_-reinforced nanocomposites compared to neat PBSA and (**b**) CNFs-reinforced nanocomposites compared to neat PBSA (DTA as dotted line).

Moreover, the thermal stability of the materials was assessed via TGA. The corresponding results are summarized in Table 3, where $T_{5\%}$ represents the temperature corresponding to 5% weight loss of the samples (commonly considered the degradation onset), $T_{50\%}$ represents the temperature corresponding to 50% weight loss, and $T_{max}$ represents the temperature corresponding to the maximum rate of weight loss, corresponding to the peak of the first derivative of the thermogram. Regarding the values for $T_{5\%}$, it can be appreciated that the presence of SiO_2_ does not significantly alter thermal stability; this is expected, since nano SiO2 is known to start degrading over 300 °C, leaving a high level of residue (over 95% by weight) [50], whereas CNFs tend to slightly decrease the onset temperature of the thermal degradation. CNFs notably start degrading after 200 °C [51] due to the organic nature of cellulose and the apparent porosity induced by the scarce compatibility of filler and matrix, as revealed by SEM investigation. The most pronounced decrease was observed for P5C, with the $T_{5\%}$ dropping from 358.2 °C for neat PBSA to 345.3 °C in the nanocomposite, corresponding to a reduction of approximately 3.7%. These results are in line with what is reported in the literature regarding similar nanocomposite systems [34,52]. Furthermore, it must be noted that all recorded values for $T_{5\%}$ are well above the maximum temperature used in the extrusion process (145 °C), thus confirming the processability of the investigated nanocomposites. From the weight loss curves in Figure 4, it can be appreciated how PBSA, P1S, P2S, and P5S all display a single-stage degradation curve, whereas the CNFs-loaded systems show initial weight loss at around 100 °C; this is likely due to the presence of bonded water, given the strongly hygroscopic nature of carboxymethylated cellulose. Moreover, this presents an additional, minor thermal degradative event around 330 °C, a temperature value which is consistent with the degradation of the cellulose filler; hence, this is attributable to the cleavage of the glycosidic bond and release of volatile compounds [46,49] (Figure 4).

### 3.3. Mechanical Properties

**Figure 5:**
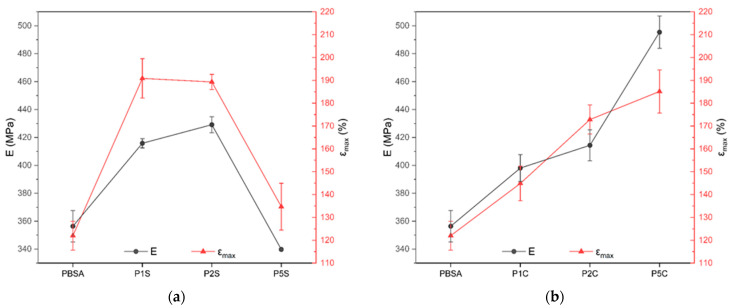
Trend of the values of E and $\varepsilon_{max}$ resulted from the tensile tests for (**a**) SiO_2_-reinforced nanocomposites compared to neat PBSA and (**b**) CNFs-reinforced nanocomposites compared to neat PBSA.

Tensile testing showed that both fillers improved the elastic modulus (E) and elongation at break ($\varepsilon_{max}$), with CNFs providing a more consistent reinforcing effect due to the favourable aspect ratio of fibres, while leaving the yielding stress ($\sigma_{y}$) and ultimate tensile stress ($\sigma_{max})$ unvaried (Figure 5, Table 4). Moreover, PBSA may interact with cellulose through hydrogen bonding by cellulose hydroxyls and bio-polyester polar groups as the ester linkages [52]. P5C provided the highest elastic modulus, with a value of 495 MPa, corresponding to an improvement of approximately 39% compared to neat PBSA. While the ductility was higher in P1S, P2S, and P5C, with values of the elongation at break of 190.9, 189.3, and 185.1%, respectively. SiO_2_ composites, which were not expected to provide significant reinforcement due to the spherical geometry of the NPs, peaked at 2 wt% in Young’s modulus (429 MPa, corresponding to an increase of 20.5%) before experiencing a significant decrease; the results of P5S were comparable with the values of neat PBSA. This result suggests that, beyond this filler content, aggregation reduced the potential strengthening effect and the efficiency of the load transfer, as well as hindering the ductile flow of the polymeric chains. Similar results were found by Ojha and Das [23] while investigating the effect of the incorporation of biogenic silica NPs in PHBV; they observed an increase in both tensile modulus and elongation at break of the nanocomposite systems, which was proportional to the filler content up to 1.5 wt%. This amount is similar to the 1 wt% and 2 wt% that we are using in this study, for E, and up to 1.0 wt% for $\varepsilon_{max}$. Above these threshold values, both E and $\varepsilon_{max}$ dropped.

The scattering recorded for both E and $\varepsilon_{max}$ remains below the threshold value of 5% in all nanocomposites, except for the case of the standard deviation of $\varepsilon_{max}$ in P5S; this result is consistent with the results of the SEM investigation, which indicated the presence of filler aggregates that locally affect the homogeneity of the material, which is particularly relevant for the 5 wt% nanofiller content.

**Figure 6:**
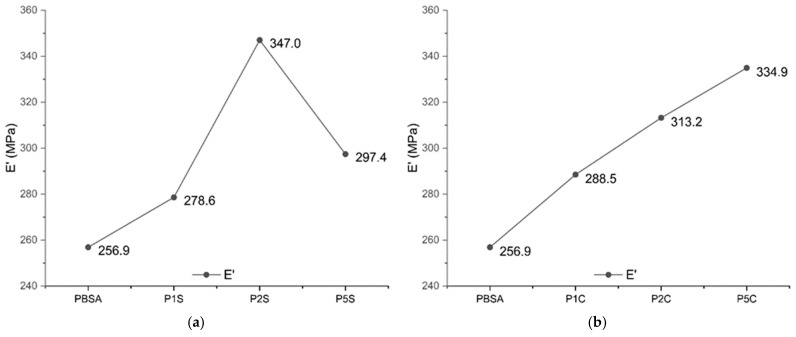
Trend for the values of the storage modulus ($E^{\prime }$) recorded via DMTA for (**a**) SiO_2_-reinforced samples compared to neat PBSA and (**b**) CNFs-reinforced samples compared to neat PBSA.

**Figure 7:**
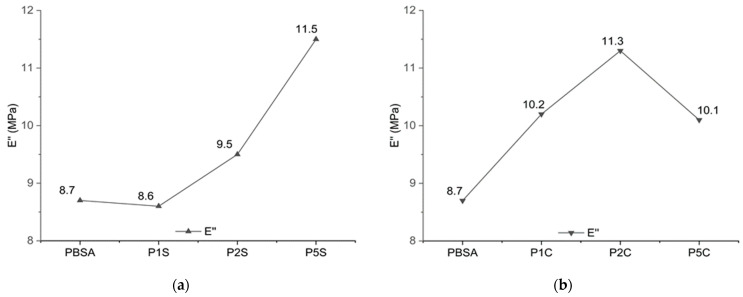
Trend for the values of the loss modulus (E″) recorded via DMTA for (**a**) SiO_2_-reinforced samples compared to neat PBSA and (**b**) CNFs-reinforced samples compared to neat PBSA.

Dynamic mechanical thermal analysis was also carried out to investigate the mechanical behaviour of the nanocomposites at different temperatures. The main results are reported in Figure 6 and Figure 7.

At 20 °C, the main contribution to the viscoelastic behaviour of the tested materials is represented by the storage modulus ($E^{\prime }$), which increased in all nanocomposite formulations, indicating enhanced stiffness, as revealed by tensile tests (Table 4). The same trend extrapolated from the data of the quasi-static tensile tests were observed in the DMTA results, as can be seen in Figure 6. In fact, for nanosilica-reinforced samples, $E^{\prime }$ peaked at 347.0 MPa with 2 wt% loading, suggesting optimal reinforcement at this concentration, while a higher amount of SiO_2_ NPs (i.e., 5 wt%) led to a deviation from the rising trend, confirming the negative effect of filler aggregates at this concentration. This finding aligns with the SEM investigation, which revealed the presence of aggregates in the formulations containing 5 wt% of SiO_2_ NPs loadings, a threshold beyond which the mechanical properties begin to decline. Nanocellulose-reinforced composites instead showed a continuous increase in the storage modulus, $E^{\prime }$, reaching 334.9 MPa at the highest filler content (5 wt%). Thus, in spite of fibres aggregation, the nanofibres still confer a strengthening effect even at 5 wt%, supporting the possible compatibility effect of hydrogen bonding between PBAS polar groups and cellulose hydroxyls [52].

The loss modulus in Figure 7 reports the trend of the peaks for energy dissipation, with an increase with increasing nanocellulose content, while for higher content of nanofibres we can observe a lowering of the Loss Modulus due to nanocellulose bundles and loss of dissipation ability.

### 3.4. Water Contact Angle and Barrier Properties

The surface characteristics, like hydrophobicity or hydrophilicity, are very important properties for polymeric materials. Typically, a hydrophobic surface is desired to prevent liquid accumulation, while a hydrophilic surface is needed for the printability and adhesion of inks. Thus, the evaluation of the surface hydrophobicity/hydrophilicity is of interest, especially for packaging applications. The most common way to evaluate the hydrophilicity/hydrophobicity of a surface is by measuring the water contact angle (WCA). Hydrophilic surfaces exhibit WCA lower than 90°, indicating a strong affinity between the material and water, which allows the droplet to spread across the surface. On the contrary, when the affinity is low, the interfacial contact is minimal and the WCA exceeds 90°, reflecting a hydrophobic behaviour.

As can be observed in Table 5, neat PBSA showed a WCA of 72.4 ± 1.8°, indicating a slight hydrophilicity. Concerning the samples incorporating the silica nanoparticles, P1S and P5S exhibited a higher WCA (73.3 ± 2.5° and 74.7 ± 3.1°, respectively) compared to neat PBSA. In these cases, the nanocomposite showed a slight increase in hydrophobicity, probably due to the hydrophobic modification of the silica nanoparticles. An opposite effect was recorded for P2S, which showed a slightly lower WCA (70.1 ± 1.4°). Nevertheless, in all cases, the variations in WCA did not exceed 3.2% and could be considered within the experimental uncertainty; thus, we conclude that the presence of SiO_2_ NPs did not significantly alter the material surface characteristics.

On the contrary, all the formulations containing CNFs displayed a smaller WCA than PBSA, due to the hydrophilic nature of CNFs enhanced by the carboxymethylation modification. Compared to SiO_2_ NPs, CNFs led to a greater modification of the WCA, ranging from 4.3 to 7.7%. It is interesting to observe that the highest variation (7.6%) was observed in the sample with the lowest content of CNFs. This can be related to the better dispersion of 1% of the nanofibrils compared to 2 and 5 wt%, as revealed by the SEM micrographs (see Section 3.1).

**Figure 8:**
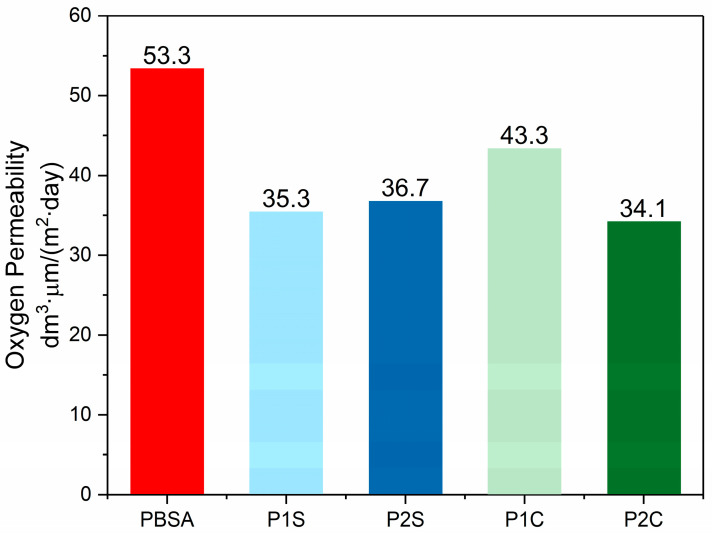
Oxygen permeability test results.

The oxygen permeability tests revealed significant improvements in barrier properties when either filler type is added to the PBSA polymeric matrix. The barrier performances were evaluated by means of the oxygen permeability (OP), measured in Figure 8. Since the mechanical investigation revealed that the samples containing 1 and 2 wt% of each filler provided the best performances and sample homogeneity, OP tests were performed solely on PBSA, P1S, P2S, P1C, and P2C.

P1S, P2S, and P2C samples showed similar OP values, reduced by over 35% when compared to neat PBSA. From a more general standpoint, the reinforced composites exhibit lower permeability to O_2_ than the unfilled material, indicating an overall improvement in barrier properties that is attributed mainly to the tortuous path effect induced by the presence of the nanofillers [18,53]. The crystallinity degrees of the films utilized for the permeability measurements were indeed similar, as indicated by thermal characterization, which proves that the barrier properties of the nanocomposites are mainly governed by the nanofillers presence. Analogous results were reported by Venkatesan and Rajeswari for PBAT reinforced with 1, 3, 5, 7, and 10 wt% of nanosilica [21]. The slightly higher oxygen permeability value displayed by the P1C sample can be rationalized by considering the voids around the CNFs fibres, which in this sample are present in a lower percentage with respect to the P2C sample, and therefore with a lower effect on the gas diffusion path.

### 3.5. Disintegration in Composting Conditions

The characteristic of PBSA of being highly biodegradable is an important property for PBSA-based materials; it is thus relevant to verify that filler addition does not hinder the compostability in the nanocomposites. Considering that PBSA is certified as an industrial compostable, the evaluation of the disintegration in compost was sufficient to assess the compostability of the developed nanocomposites.

**Figure 9:**
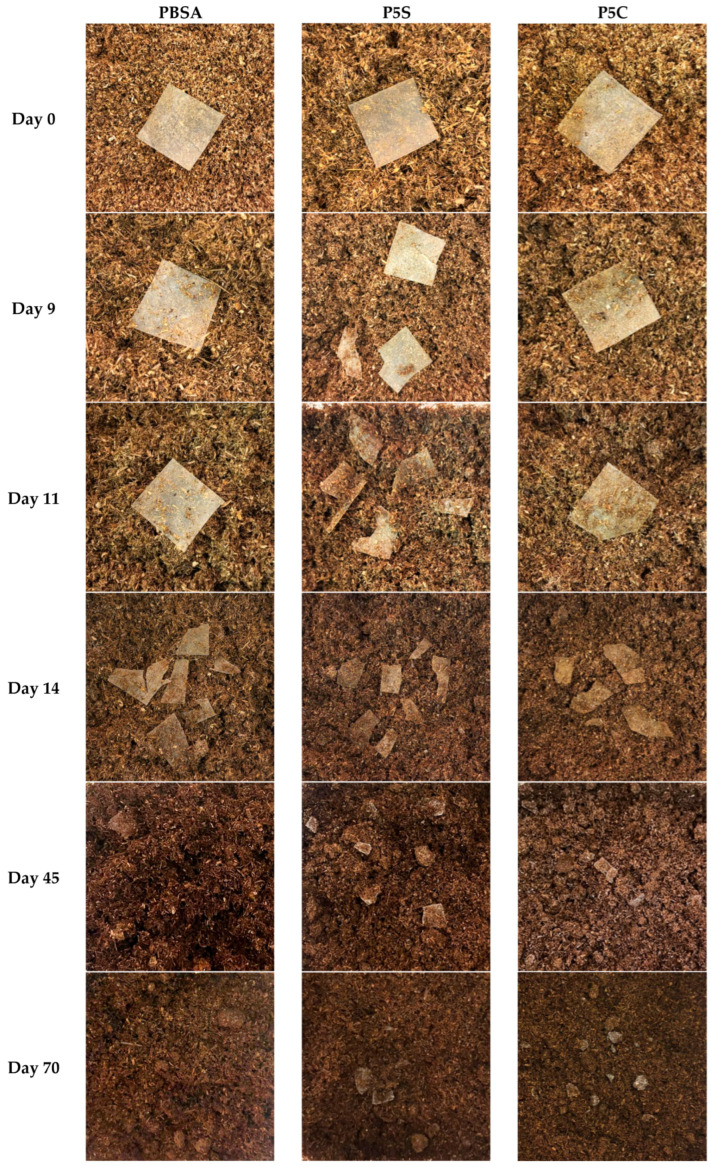

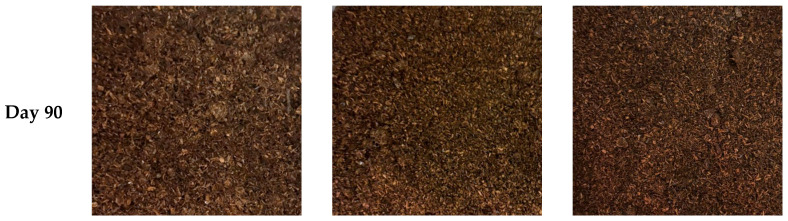
Advancements of the disintegration under composting conditions of PBSA, P5S, and P5C film-like samples.

P5S and P5C were selected to evaluate the disintegration under composting conditions, as it was expected that these formulations, being the ones containing the greatest filler fraction (5 wt%), would be able to provide the most representative results. Neat PBSA was also analysed as the reference material. All the materials were prepared in film-like samples with a thickness of 85–100 μm and buried in a simulated compost substrate according to the method reported in ISO 20200. The synthetic compost presented a regular evolution in colour, odour, and chemical parameters, which were regularly monitored in agreement with the ISO 20200 procedure [44]. At regular time frames, the samples were retrieved, photographed, and monitored for deterioration. Visual representations of the results can be seen in Figure 9.

The results of the test showed accelerated degradation in the nanocomposites compared to the neat PBSA polymeric matrix, which is consistent with findings reported in the literature for composites and nanocomposites of bio-polyesters loaded with similar fillers [29,30,33]. Specifically, P5C films showed fragmentation as early as day 8 from the start of the test, due to their hydrophilic nature and inherent hygroscopicity of cellulose, and P5S test fragments also degraded faster than the neat matrix, with the onset of fragmentation recorded around day 11, likely due to increased surface area and porosity. Neat PBSA started visible fragmentation on day 14. After 90 days from the start of the test, the contents of the reactors were dried in an oven at 58 °C until a constant weight was reached (approximately 10 days), then sieved through a 2 mm mesh, according to the standard ISO 20200 [44].

As no residues attributable to the polymer were detected after the sifting process, it could be concluded that all samples completed disintegration within 90 days. Hence, given the positive results of the disintegration test, as per regulation ISO 17088:2021, the OK compost certification of PBSA can be extended to its SiO_2_ NPs-based nanocomposites and its CNF-based nanocomposites [44,54,55].

## 4. Conclusions

This study deepened the investigation into PBSA nanocomposites incorporating SiO_2_ nanoparticles or cellulose nanofibrils (CNFs); we considered the advantages of this polymeric matrix: it is highly biodegradable in several environments, and it is flexible and easy to process. The nanocomposites had been realised in the absence of any compatibilizer in order to focus on the effect of content and morphology of the nanofiller on morphological, thermal, mechanical, and barrier properties of PBSA-based nanocomposites.

Thermal characterization revealed that the thermal properties of the polymer matrix, such as glass transition temperature (T_g_) and melting temperature (T_m_), were not altered by the presence of the nanofillers. In addition, although nanocomposites containing CNFs showed a slightly reduced T_5%_, due to the degradation of the cellulose fibrils, all the investigated nanocomposites showed a degradation temperature well above the processing temperature employed during the extrusion (145 °C); thus, the results confirm the thermal stability of these PBSA-based nanocomposites under the applied manufacturing conditions.

Concerning the mechanical performance, both fillers contributed to an overall increase in elastic modulus and elongation at break with increasing filler content (except for formulation P5S). As expected, CNFs provided superior reinforcement with an increase in the Young modulus up to 39%, due to the favourable aspect ratio.

Since the materials could potentially be used for packaging applications, the barrier properties and water contact angle (WCA) were evaluated. From the results, a significant reduction in oxygen permeability was observed when 1 and 2% of nanofillers were added to the matrix. Moreover, the SiO_2_-reinforced composites showed a WCA ranging from 70 to 75°, very similar to the WCA value of neat PBSA (72°). Thus, indicating that the presence of this filler did not significantly alter the surface hydrophilicity/hydrophobicity of the nanocomposites. Indeed, the presence of CNFs in the nanocomposites slightly decreased the WCA, which was in the range between 67 and 69°, indicating a modest enhancement in surface hydrophilicity related to the hydrophilic nature of CNFs.

The disintegration tests under composting conditions revealed that the presence of both nanofillers preserved the compostability of the matrix and appeared to accelerate the onset of the disintegration process. In other words, the biodegradability, that is, the most important property of PBSA-based materials, was not affected by the nanofillers.

In conclusion, the present study demonstrates that PBSA-based nanocomposites containing SiO_2_ nanoparticles and cellulose nanofibrils are promising candidates for single-use, large volume applications such as packaging or agriculture. Nevertheless, future work should explore the use of compatibilizers to improve filler dispersion and interfacial adhesion, as well as investigate alternative filler morphologies (e.g., platelets or lamellae) to further enhance barrier and mechanical properties.

## Figures and Tables

**Table 1:** DSC1 results.

Sample	$T_{c}$ (°C)	$T_{m,I}$ (°C)	$T_{m,II}$ (°C)	$T_{m,III}$ (°C)	$\Delta H_{m}$ (J/g)	$X_{c}$ (%)
PBSA	52.8 ± 0.4	56.6 ± 0.5	81.4 ± 0.7	87.0 ± 0.6	95.4 ± 0.2	48 ± 0.4
P1S	52.6 ± 0.3	54.4 ± 0.3	81.8 ± 0.6	87.0 ± 0.4	96.4 ± 0.5	48 ± 0.3
P2S	53.1 ± 0.2	57.4 ± 0.4	80.8 ± 0.5	87.0 ± 0.5	94.5 ± 0.3	47 ± 0.4
P5S	51.4 ± 0.3	57.2 ± 0.2	81.1 ± 0.7	87.6 ± 0.6	95.5 ± 0.3	48 ± 0.5
P1C	51.8 ± 0.4	58.2 ± 0.5	80.9 ± 0.5	87.6 ± 0.5	96.8 ± 0.4	48 ± 0.3
P2C	51.5 ± 0.2	55.9 ± 0.4	79.8 ± 0.4	87.0 ± 0.6	98.3 ± 0.3	49 ± 0.5
P5C	49.9 ± 0.1	55.1 ± 0.5	80.6 ± 0.5	87.8 ± 0.3	101.8 ± 0.5	51 ± 0.6

**Table 2:** Comparison between the $T_{g}$ values obtained via DSC2 and DMTA analysis.

Sample	$T_{g}^{DSC}$ (°C)	$T_{g}^{DMTA}$ (°C)
PBSA	−46.9 ± 0.2	−47.5 ± 0.2
P1S	−47.3 ± 0.4	−46.1 ± 0.1
P2S	−46.9 ± 0.2	−46.3 ± 0.3
P5S	−46.9 ± 0.3	−46.3 ± 0.3
P1C	−46.5 ± 0.1	−47.1 ± 0.3
P2C	−45.9 ± 0.1	−46.3 ± 0.1
P5C	−46.2 ± 0.2	−47.1 ± 0.3

**Table 3:** TGA results.

Sample	$T_{5\%}$ (°C)	$T_{50\%}$ (°C)	$T_{max}$ (°C)
PBSA	358.2 ± 0.2	399.9 ± 0.2	407.5 ± 0.1
P1S	360.4 ± 0.1	401.7 ± 0.1	408.0 ± 0.2
P2S	359.7 ± 0.3	400.3 ± 0.2	404.5 ± 0.2
P5S	364.3 ± 0.2	407.0 ± 0.3	409.5 ± 0.1
P1C	354.1 ± 0.2	398.1 ± 0.2	403.4 ± 0.2
P2C	352.2 ± 0.3	396.2 ± 0.1	401.9 ± 0.3
P5C	345.3 ± 0.1	395.9 ± 0.3	400.6 ± 0.2

**Table 4:** Results of the tensile tests: elastic modulus (E), yielding stress ($\sigma_{y}$), ultimate tensile stress ($\sigma_{max})$, and elongation at break ($\varepsilon_{max}$).

Sample	E (MPa)	$\sigma_{y}$ (MPa)	$\sigma_{max}$ (MPa)	$\varepsilon_{max}$ (MPa)
PBSA	356.4 ± 22.5	18.4 ± 0.3	25.5 ± 0.7	122.0 ± 19.2
P1S	415.8 ± 6.7	17.9 ± 0.1	25.4 ± 0.6	190.9 ± 17.3
P2S	429.1 ± 11.5	17.8 ± 0.3	24.3 ± 0.7	189.3 ± 6.6
P5S	339.8 ± 0.3	18.2 ± 0.5	21.5 ± 2.7	134.7 ± 41.0
P1C	398.1 ± 19.4	18.8 ± 0.5	26.5 ± 0.9	144.9 ± 15.3
P2C	414.4 ± 22.4	17.3 ± 1.4	25.0 ± 1.5	172.8 ± 12.7
P5C	495.4 ± 23.3	17.1 ± 0.3	23.1 ± 0.8	185.1 ± 18.9

**Table 5:** Water contact angle (WCA) measurement results.

Sample	WCA (°)
PBSA	72.4 ± 1.8
P1S	73.3 ± 2.5
P2S	70.1 ± 1.4
P5S	74.7 ± 3.1
P1C	66.9 ± 3.2
P2C	69.4 ± 2.8
P5C	68.1 ± 3.0

## Data Availability

The original contributions presented in this study are included in the article. Further inquiries can be directed to the corresponding author.
